# Solar-Driven TiO_2_ Photocatalytic Degradation of Live Chemical Warfare Agents: Performance Evaluation and Mechanistic Analysis

**DOI:** 10.3390/molecules31132227

**Published:** 2026-06-24

**Authors:** Sungki Kim, Doo-Hee Lee, Myungsik Shin, Jin Kim, Min-Kun Kim, Ku Kang

**Affiliations:** 1 CBRN Defense Research Institute (CDRI), Ministry of National Defense, Seoul 06796, Republic of Korea; 2 Radioisotope Research Division, Korea Atomic Energy Research Institute (KAERI), Daejeon 17058, Republic of Korea; 3 Department of Materials Science and Engineering, Myongji University, Yongin 17058, Republic of Korea

**Keywords:** chemical warfare agents, hazardous organic contaminants, TiO_2_ photocatalysis, solar-assisted decontamination, nitrogen mustard, degradation mechanism, high-resolution mass spectrometry, green chemistry

## Abstract

The environmentally sustainable decontamination of chemical warfare agents (CWAs) remains a critical challenge. This study reports the solar-driven photocatalytic degradation of live CWAs—GD, HD, HN1, and HN2—using titanium dioxide (TiO_2_) under natural sunlight. Experiments were conducted in an OPCW-designated laboratory to ensure authenticity and practical relevance. TiO_2_ exhibited substantial photocatalytic activity, achieving 60% degradation of GD, 63% of HD, 76% of HN1, and 93% of HN2 after 6 h. High-resolution mass spectrometry (HR-MS) analysis suggested plausible degradation pathways for nitrogen mustards consistent with the higher apparent reactivity of HN2; detailed identification of intermediates and reactive oxygen species remains a subject for future investigation. These findings provide mechanistic insights into the photocatalytic behavior of nitrogen-based agents and address a notable gap in studies that have largely focused on sulfur mustards and nerve agents. Beyond military applications, this solar-assisted photocatalytic approach provides mechanistic information relevant to the green remediation of highly toxic organic contaminants and broader chemical hazard mitigation. This work contributes foundational knowledge toward eco-friendly decontamination technologies capable of mitigating diverse CWA threats.

## 1. Introduction

Chemical warfare agents (CWAs) continue to pose severe threats to human safety and environmental security due to their high toxicity, persistence and potential misuse in warfare or terrorist activities. The development of efficient and environmentally friendly decontamination technologies remains a critical issue worldwide [1,2]. Conventional methods, such as incineration, alkaline hydrolysis, or chemical neutralization, often lead to secondary environmental hazards and require extensive logistical support [3,4,5]. Consequently, there has been growing interest in developing alternative methods that minimize environmental impacts, while efficiently degrading highly toxic CWAs under mild conditions [6,7,8].

Among various approaches, photocatalytic degradation has emerged as a promising strategy due to its effectiveness in mineralizing hazardous organic compounds into non-toxic products using renewable energy sources such as solar light [9,10]. Titanium dioxide (TiO_2_), in particular, has attracted significant attention as a robust, cost-effective, and environmentally benign photocatalyst [11,12]. Recent studies have extensively demonstrated the capability of TiO_2_ to degrade various environmental pollutants and chemical threats [13,14,15]. However, the majority of previous studies have utilized simulants or model compounds instead of actual live CWAs due to the stringent regulations, high toxicity, and complex handling procedures involved [16,17,18]. Thus, data derived from simulants may not adequately represent the actual photocatalytic performance against genuine agents. Recent reports on engineered heterojunction photocatalysts further illustrate the applicability of this strategy to environmental pollutant remediation [19,20].

While extensive literature has documented the photocatalytic degradation of nerve agents (G-series, such as GD and GB) and sulfur mustard (HD)—largely driven by their historical usage and relatively easier accessibility for research [21,22,23]—significantly fewer studies have addressed nitrogen mustard agents (HN series). This research gap is primarily attributed to the substantial synthetic challenges, higher intrinsic reactivity, and greater analytical complexity of HN compounds compared to their sulfur-based counterparts [24,25]. Although degradation kinetics, reaction mechanisms, and catalyst optimization strategies have been well established for nerve agents and sulfur mustard [21,23], the few existing studies on nitrogen mustards are largely indirect, relying on simulants or reporting only bulk degradation rates without detailed mechanistic elucidation [24,26].

Nitrogen mustards, due to their bifunctional alkylating structure and rapid hydrolytic reactivity, pose unique degradation challenges that differ substantially from sulfur-based CWAs. Their complex decomposition behavior under photocatalytic conditions remains poorly characterized, and no systematic studies to date have comprehensively addressed the identification of intermediates, reaction pathways, or kinetic profiles. This lack of detailed mechanistic understanding leaves a critical knowledge gap in environmental chemical engineering and decontamination research, limiting the development of targeted photocatalytic strategies capable of efficiently neutralizing nitrogen-based CWAs under realistic conditions [27,28].

Addressing this gap is essential for advancing both scientific understanding and the practical application of solar-driven photocatalytic decontamination technologies. These studies require specialized laboratory infrastructure and considerable expertise, typically available only in laboratories designated by international regulatory bodies such as the Organization for the Prohibition of Chemical Weapons (OPCW). This ensures rigorous handling and experimental protocols, allowing accurate assessment of photocatalytic degradation performance against actual CWAs [29,30].

In this context, the present study aims to investigate the solar-driven photocatalytic degradation of live chemical warfare agents, specifically GD, HD, and the rarely studied nitrogen mustard agents, HN1 and HN2, using TiO_2_ as a catalyst. Conducted in an OPCW-designated laboratory, this research utilizes actual live agents to ensure the practical relevance and authenticity of the results. The objectives of this study are: (1) to quantitatively assess the degradation performance of TiO_2_ under environmentally relevant solar irradiation conditions; (2) to obtain qualitative mechanistic information on plausible transformation pathways of the nitrogen mustard agents (HN1 and HN2) using high-resolution mass spectrometry (HR-MS); (3) to explore the multi-sectoral application potential of this photocatalytic approach, extending beyond military applications to industrial, environmental, and national security domains. Ultimately, the findings from this work will provide foundational insights for developing eco-friendly, solar-assisted decontamination technologies capable of mitigating diverse chemical threats.

## 2. Results

### 2.1. Photocatalytic Degradation of GD and HD

To evaluate the photocatalytic degradation performance of titanium dioxide (TiO_2_) under solar irradiation, experiments were conducted using GD and HD as representative chemical warfare agents. Control experiments without TiO_2_ (sunlight only) or without sunlight (dark conditions) were performed simultaneously to verify the necessity of both the catalyst and light activation.

The degradation results after 6 h are summarized in Table 1. Under dark conditions, the residual concentration of GD and HD remained above 89%, indicating negligible degradation.

Similarly, sunlight exposure without TiO_2_ did not result in significant decomposition. In contrast, when TiO_2_ was present under sunlight, GD showed approximately 60% degradation and HD exhibited approximately 63% degradation after 6 h.

**Figure 1:**
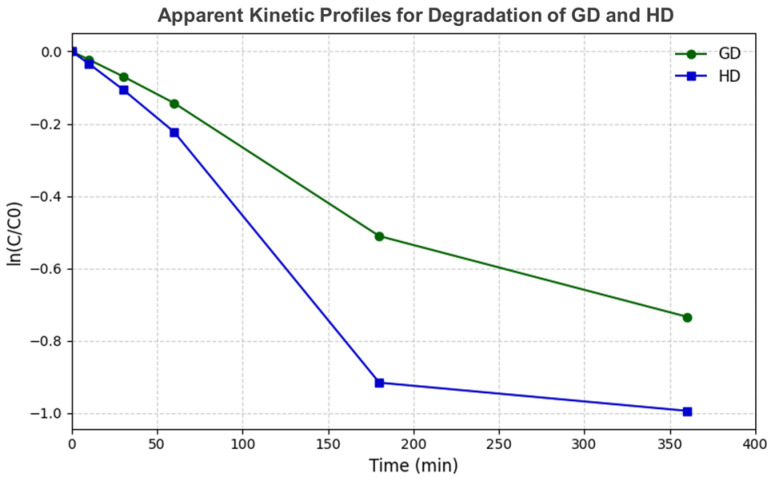
Apparent kinetic profiles for the TiO_2_-mediated degradation of GD and HD under solar irradiation. Data are presented for comparative visualization only; the corresponding rate values are endpoint-derived apparent estimates rather than statistically validated pseudo-first-order kinetic constants.

The kinetic behavior of GD and HD under photocatalytic conditions was further investigated assuming a pseudo-first-order model. As shown in Figure 1, the ln(C/C_0_) versus reaction time profiles are approximately consistent with an apparent pseudo-first-order framework; however, because the GD and HD datasets are based on a limited number of time points, the resulting values should be regarded as endpoint-derived apparent estimates rather than as definitive kinetic constants. The endpoint-derived apparent rate estimates indicated that HD showed slightly greater parent-agent loss than GD under the tested TiO_2_ + sunlight condition. These values are used only for comparative ranking under the present experimental window and should not be interpreted as definitive kinetic constants. Detailed kinetic parameters are discussed in the following section.

### 2.2. Photocatalytic Degradation of HN1 and HN2

Photocatalytic degradation experiments were extended to nitrogen mustard agents HN1 and HN2, which are known for their higher chemical reactivity and analytical challenges compared to sulfur mustards [24,25]. Control experiments were conducted to isolate the effects of sunlight and TiO_2_ catalyst.

**Figure 2:**
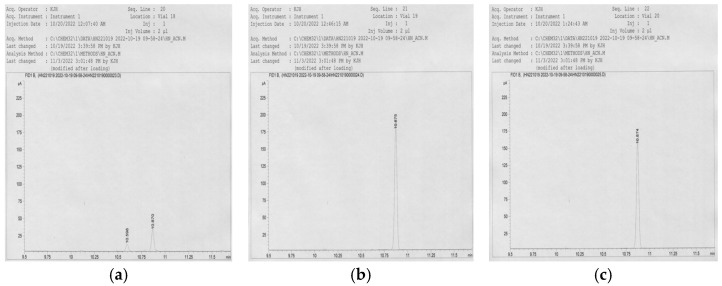
GC-FID chromatograms of HN1 after 6 h under different experimental conditions: (**a**) TiO_2_ + sunlight, (**b**) sunlight only, (**c**) TiO_2_ only (dark condition).

GC-FID chromatograms of HN1 after 6 h under different experimental conditions are presented in Figure 2. When both TiO_2_ and sunlight were present (Figure 2a), a significant reduction in the HN1 peak at retention time (RT) 10.870 min was observed. In contrast, the samples exposed to sunlight only (Figure 2b) or TiO_2_ only under dark conditions (Figure 2c) exhibited minimal changes in peak intensity, indicating that both photocatalyst and solar irradiation are essential for effective degradation.

**Figure 3:**
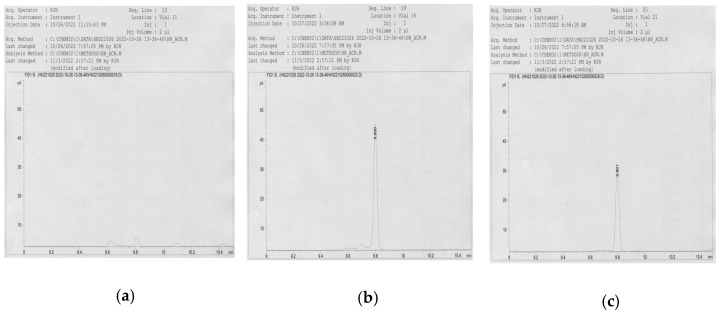
GC-FID chromatograms of HN2 after 6 h under different experimental conditions: (**a**) TiO_2_ + sunlight, (**b**) sunlight only, (**c**) TiO_2_ only (dark condition).

**Figure 4:**
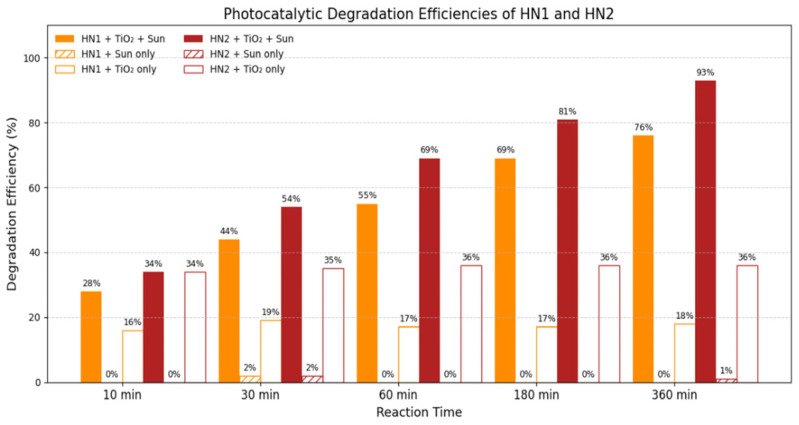
Photocatalytic degradation efficiencies of HN1 and HN2 under different reaction conditions and reaction times.

Similarly, GC-FID chromatograms of HN2 after 6 h of reaction are presented in Figure 3. A pronounced reduction of the HN2 peak was evident only under the combined TiO_2_ and sunlight condition, further supporting the synergistic necessity of catalyst and light activation. The degradation efficiencies of HN1 and HN2 as a function of reaction time and experimental conditions are summarized in Figure 4. Under TiO_2_ and sunlight exposure, HN1 exhibited a degradation efficiency of 76%, while HN2 achieved 93% after 6 h. In contrast, negligible degradation was observed for the control groups.

**Figure 5:**
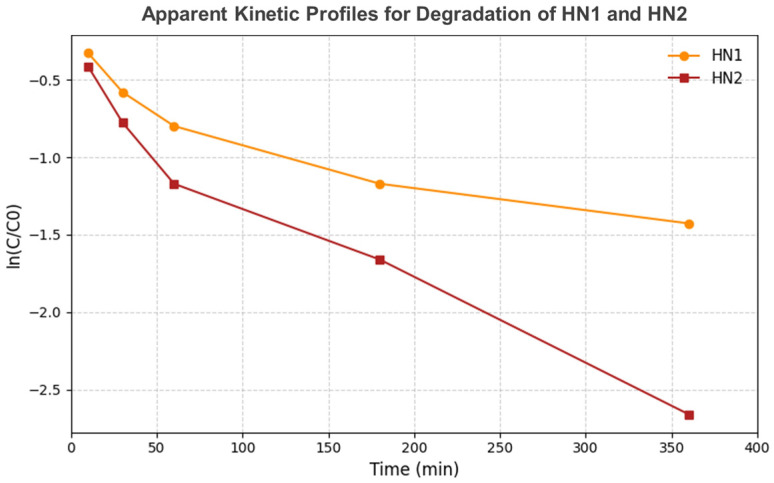
Apparent kinetic profiles for the TiO_2_-mediated degradation of HN1 and HN2 under solar irradiation. The profiles are used for comparative visualization; the reported rate values should be interpreted as endpoint-derived apparent estimates.

The photocatalytic degradation kinetics of HN1 and HN2 were further analyzed assuming pseudo-first-order behavior. The ln(C/C_0_) versus time plots are presented in Figure 5. The calculated rate constant for HN2 was higher than that for HN1, consistent with the observed degradation efficiencies. As with GD and HD, these values are reported as endpoint-derived apparent estimates intended for comparative ranking of the agents under the same experimental window, and do not constitute statistically powered pseudo-first-order rate constants.

For comparative evaluation, endpoint-estimated apparent rate constants were calculated from the degradation efficiencies at 360 min and are summarized in Table 2. Among the tested agents, HN2 exhibited the highest apparent rate constant, followed by HN1, HD, and GD. This trend is consistent with the degradation efficiencies observed under TiO_2_ + sunlight conditions. We emphasize that the values reported in Table 2 are endpoint-derived apparent estimates calculated from the 360 min residual concentration values; they are intended for cross-agent comparison under identical solar exposure rather than for absolute kinetic interpretation. The present dataset, obtained under OPCW-regulated live-agent constraints, was not designed to support full pseudo-first-order regression with replicate statistics and confidence intervals; controlled-irradiation follow-up experiments will be required for that purpose.

The apparent rate constants were estimated from the 360 min degradation efficiencies using *k_app_ = −ln (C/C*_0_*)/t*. As noted above, these values are reported as endpoint-derived apparent estimates intended for comparative ranking among the tested agents rather than as definitive pseudo-first-order rate constants.

Overall, these results indicate substantial parent-agent loss for nitrogen mustard agents, particularly HN2, under TiO_2_-mediated solar photocatalytic conditions. The combined presence of catalyst and light activation appears to be a necessary condition for the parent-agent degradation observed under the present experimental window.

### 2.3. Degradation Mechanism Analysis

The degradation mechanisms of nitrogen mustard agents (HN1 and HN2) under photocatalytic conditions were further investigated through both gas chromatography (GC-FID) and high-resolution mass spectrometry (HR-MS) analyses. Reaction-stage signals consistent with potential intermediates and end-products were observed and were used, together with established TiO_2_ photocatalysis chemistry, to propose plausible mechanistic pathways.

GC-FID chromatograms after 6 h of photocatalytic reaction (Figure 2 and Figure 3) revealed significant differences depending on the experimental conditions. For HN1, negligible degradation was observed under sunlight-only or TiO_2_-only (dark) conditions, with approximately 17% degradation. However, when both TiO_2_ and sunlight were present, the degradation efficiency reached 76%. This confirms that the combination of photocatalyst and light activation is critical for effective HN1 decomposition. Similar trends were observed for HN2, exhibiting a higher overall degradation efficiency of 93%.

The residual loss observed under dark and sunlight-only controls is not negligible and likely reflects the combined contribution of (i) spontaneous hydrolysis of the chloroethyl arms in the acetonitrile/water (99:1) solvent—a known background process for nitrogen mustards—(ii) physical adsorption of the parent agent on the TiO_2_ surface in the dark control, and (iii) a small volatilization loss across the vial seal during the 6 h irradiation window. PTFE/silicone septa and constant headspace were used to minimize the volatilization contribution. A quantitative mass-balance separation among these processes was outside the scope of the present proof-of-concept campaign and is identified as future work. The substantial enhancement observed only under the combined TiO_2_ + sunlight condition is therefore attributed primarily to photocatalytic degradation rather than to hydrolysis or sorption.

**Figure 6:**
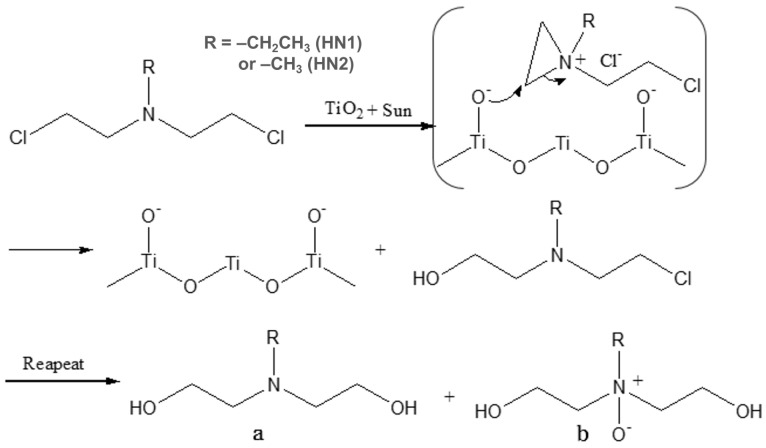
Proposed photocatalytic degradation mechanisms of HN1 and HN2 under solar irradiation using TiO_2_ catalyst. In the proposed structures, R denotes CH_2_CH_3_ for HN1 or CH_3_ for HN2; a and b indicate the proposed intermediate species shown in the pathway.

Subsequent HR-MS analysis of the HN1 and HN2 samples provided qualitative mechanistic information. For HN2, the HR-MS observations are consistent with a plausible degradation pathway involving sequential dechlorination and hydrolysis under photocatalytic oxidation, as illustrated in Figure 6. For HN1, the HR-MS observations are likewise consistent with an initial loss of chloroethyl groups followed by oxidative transformation of the amine moiety. Figure 6 summarizes a plausible photocatalytic degradation pathway for HN1 and HN2 based on the observed decrease in parent-agent signals, the available HR-MS observations, and established TiO_2_ photocatalysis chemistry. The pathway begins with photogeneration of surface-bound hydroxyl radicals on the TiO_2_ photoexcited state, which abstract a chloride from one of the 2-chloroethyl arms; a second analogous step on the remaining arm, combined with hydrolytic substitution, yields a bis-hydroxyethyl tertiary amine. Subsequent oxidative cleavage of one of the C–N bonds, also attributable to reactive oxygen species, produces the corresponding aldehyde and a secondary amine. For HN1, the same dechlorination → hydroxyl substitution → oxidative C–N cleavage sequence is proposed but is sterically retarded by the bulkier N-ethyl substituent. We emphasize, however, that the present study does not provide a complete intermediate assignment table, extracted-ion chromatograms, product-ion spectra, or radical-scavenger experiments; consequently, the pathway depicted in Figure 6 should be interpreted as a working mechanistic hypothesis rather than a fully confirmed reaction sequence.

Overall, the combined GC-FID and HR-MS observations are consistent with a plausible degradation pathway involving photocatalytically induced hydrolysis, dechlorination, and oxidative cleavage of nitrogen-mustard bonds; this pathway is presented as a working mechanistic hypothesis. The higher degradation efficiency of HN2 compared to HN1 is likely attributed to structural variations, such as reduced steric hindrance due to the smaller methyl substituent in HN2 compared to the ethyl group in HN1, facilitating more efficient radical attack and degradation. This interpretation is qualitatively consistent with the smaller three-dimensional profile of the N-methyl substituent in HN2 relative to the N-ethyl group in HN1, which is expected to reduce shielding of the nitrogen lone pair and facilitate the initial interaction with photogenerated hydroxyl radicals. The present study does not directly probe this steric argument by DFT or radical-scavenger experiments, and the steric-hindrance interpretation is therefore presented as a working hypothesis rather than a confirmed mechanistic conclusion.

### 2.4. Practical Implications for Decontamination Applications

The results presented in this study highlight significant potential for TiO_2_-based photocatalytic systems as candidate components for sustainable CWA decontamination strategies. The efficient degradation of GD, HD, HN1, and HN2 demonstrated herein indicates the promising applicability of these photocatalytic systems in both passive and active decontamination scenarios.

TiO_2_ photocatalysts can be integrated into various self-cleaning and self-decontaminating materials, such as coatings on infrastructure, vehicles, textiles, and personal protective equipment, effectively reducing hazards associated with exposure to toxic CWAs [24,26,29]. Photocatalytic coatings have previously shown efficacy in environmental remediation applications, including air and water purification, by utilizing ambient solar radiation to promote sustained oxidative reactions [5,21,30,31]. Thus, these materials hold potential not only for military defense scenarios but also in broader industrial and civilian contexts [10,11,17,18].

Military applications specifically can benefit greatly from such technology. The integration of photocatalytic surfaces on military equipment and installations can passively degrade residual CWAs, thereby reducing the logistic and environmental burdens associated with traditional chemical neutralization methods [14,15,17,18]. Furthermore, stationary installations incorporating photocatalytic materials could serve as continuous decontamination points, enhancing protection in sensitive locations such as military bases or critical national infrastructure [10,15].

Industrial and environmental applications extend beyond military contexts. TiO_2_-based photocatalysis has been reported to be effective for degrading persistent organic pollutants and hazardous materials in industrial waste streams, underscoring its versatility and relevance to industrial safety and environmental protection [25,32,33,34]. Additionally, the use of photocatalytic coatings could significantly mitigate environmental impacts resulting from accidental chemical spills or industrial incidents by providing a passive, sustainable remediation method [35].

Despite these advantages, the practical implementation of photocatalytic technologies for immediate emergency response remains limited due to relatively slower reaction rates compared to conventional chemical treatments [3,33]. Thus, while photocatalytic systems are ideal for passive, long-term contamination management, they may be complemented by conventional rapid-response methods in high-risk situations where immediate neutralization of hazardous substances is essential.

Overall, this study underscores the broad applicability and environmental benefits of TiO_2_ photocatalysis, positioning it as a sustainable and efficient alternative to traditional chemical decontamination approaches. Future research efforts should aim to enhance reaction efficiencies through catalyst optimization, as well as evaluate practical field-scale deployments to fully realize the potential of this promising technology [31,34,36]. It is important to stress that the experiments reported here were conducted in a homogeneous acetonitrile/water (99:1) suspension at laboratory scale and therefore constitute a proof-of-concept demonstration. They do not, by themselves, establish the performance of TiO_2_-based coatings, textiles, or field-deployable surfaces, nor do they demonstrate complete mineralization or detoxification of the parent agents; disappearance of the parent molecule, as monitored by GC-FID and HR-MS, should not be equated automatically with full detoxification. These applications are therefore discussed throughout the manuscript as prospective directions rather than as established outcomes of the present study.

## 3. Discussion

### 3.1. Effectiveness and Comparison of Photocatalytic Degradation

**Figure 7:**
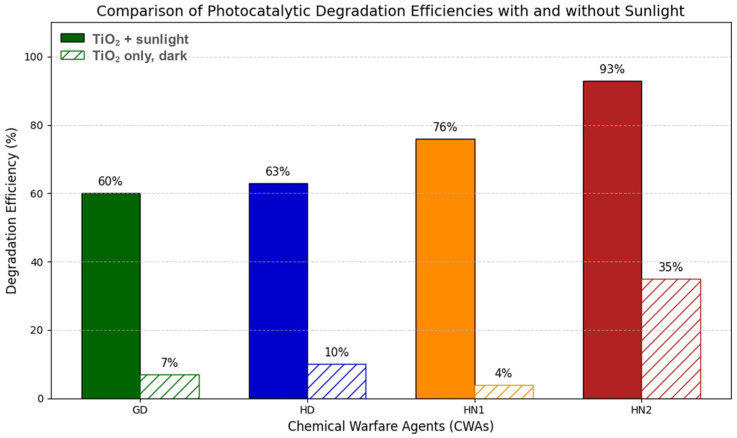
Comparison of photocatalytic degradation efficiencies of CWAs (GD, HD, HN1, and HN2) with and without sunlight. Solid-colored bars correspond to the TiO_2_ + sunlight condition; hatched bars correspond strictly to the TiO_2_-only dark control. The complementary sunlight-only (no TiO_2_) control values are reported separately in Table 1 and Figure 4 to avoid combining different controls under a single label. Green, blue, orange, and red bars indicate GD, HD, HN1, and HN2, respectively.

The present study demonstrated significant photocatalytic degradation efficiencies for the chemical warfare agents GD, HD, HN1, and HN2 under natural solar irradiation, confirming the promising applicability of TiO_2_ as a viable catalyst for environmental and security-related decontamination efforts. As illustrated clearly in Figure 7, the degradation efficiencies under solar irradiation reached 60% for GD, 63% for HD, and notably higher efficiencies for the nitrogen mustard agents, achieving 76% for HN1 and 93% for HN2 after 6 h of reaction. In contrast, under dark conditions, significantly lower degradation efficiencies were observed (4–35%), underscoring the critical role of solar activation in photocatalytic reactions.

Previous research on photocatalytic degradation of chemical warfare agents primarily involved simulants due to regulatory constraints and experimental safety considerations [16,17]. In contrast, this study employed actual CWAs, providing more realistic and practical insights into the performance of TiO_2_-mediated photocatalysis under relevant environmental conditions. Specifically, our findings related to nitrogen mustard agents (HN1 and HN2) offer novel and valuable benchmarks for future research, given that these agents have received considerably less attention in the existing literature [15,18].

A particularly notable observation in this study was the substantially higher photocatalytic degradation efficiency of HN2 compared to HN1, as summarized in Figure 7. This enhanced reactivity can be attributed to structural and steric factors unique to HN2. Chemically, HN2 possesses a methyl substituent attached to the nitrogen atom, leading to reduced steric hindrance around the reactive nitrogen center. Such structural characteristics likely facilitate more effective adsorption and subsequent oxidative interactions between the nitrogen mustard molecule and hydroxyl radicals (OH·) generated on the activated TiO_2_ surface. Indeed, previous theoretical and experimental studies have also indicated that reduced steric hindrance and favorable electronic properties significantly enhance susceptibility to photocatalytically generated radicals, thereby promoting higher degradation kinetics and efficiencies [32]. The participation of hydroxyl radicals is invoked here on the basis of the well-established TiO_2_ photocatalytic literature and is consistent with the observed dechlorination and oxidative cleavage pattern; however, the present study did not include radical-scavenger or spin-trap experiments, and the explicit assignment of reactive-oxygen-species roles is therefore presented as a literature-supported hypothesis rather than an experimentally confirmed mechanism for these substrates.

Furthermore, the degradation performance observed for sulfur mustard (HD) in this study (approximately 63%) aligns closely with previously reported experimental findings [17,18,33], affirming the reliability and reproducibility of our experimental conditions. In contrast, the moderate degradation efficiency observed for the nerve agent GD highlights potential areas for catalyst optimization, including metal doping, surface modification, or the incorporation of additional photocatalytically active materials. Such modifications have been extensively reported in prior literature to enhance photocatalytic performance significantly [11,12,29,31,34,36].

In summary, the current study not only validates the effectiveness of TiO_2_ photocatalysts for degrading diverse CWAs but also highlights structural considerations essential for optimizing catalytic materials. These insights underscore the need for continued research and development of advanced photocatalytic systems tailored to specific chemical threats, particularly focusing on agents with distinct structural and reactive characteristics such as the nitrogen mustards.

### 3.2. Detailed Mechanistic Insights into HN1 and HN2 Degradation

The HR-MS analysis provided qualitative mechanistic information on the transformation of HN1 and HN2 under TiO_2_-mediated solar photocatalytic conditions. Because complete intermediate-assignment tables, extracted-ion chromatograms, product-ion spectra, and ROS-scavenger experiments were not available in the present proof-of-concept campaign, the proposed pathways should be interpreted as plausible working hypotheses rather than fully confirmed mechanisms. Previous literature has highlighted the limited number of mechanistic studies available for nitrogen mustards compared to the extensively studied HD and nerve agents [15,18]. Consequently, the qualitative mechanistic information obtained in our study contributes useful observations to the field, especially due to the challenges in handling and analyzing these highly reactive substances [24,25].

The available HR-MS observations are consistent with transformation processes involving dechlorination, hydrolysis, and oxidative cleavage of nitrogen-mustard structures, with apparent differences between HN1 and HN2 that may reflect the varying steric and electronic properties of the substituents on the nitrogen atom. The proposed primary pathway is consistent with sequential dechlorination and oxidative hydrolysis processes that, in line with established TiO_2_ photocatalysis chemistry, are typically attributed to hydroxyl radicals (OH·) generated on the TiO_2_ surface under solar irradiation. Such degradation pathways are consistent with previously proposed oxidative degradation mechanisms of structurally related nitrogenous compounds under photocatalytic conditions [26,32].

Interestingly, the degradation efficiency of HN2 was notably higher (93%) compared to HN1 (76%), a difference likely arising from the distinct steric hindrance and reactivity profiles. HN2 possesses a methyl group on the nitrogen atom, leading to less steric hindrance and potentially enhanced susceptibility to oxidative attack by photocatalytically generated radicals. This aligns well with previous theoretical predictions suggesting that steric hindrance significantly influences photocatalytic reaction kinetics [32].

The HR-MS observations are consistent with the proposed mechanistic pathway, including the progressive loss of chlorine atoms and the oxidation of amine groups into structurally transformed products that may lack the bifunctional chloroethyl alkylating motif of the parent HN-series agents. This mechanistic understanding is pivotal for the targeted optimization of photocatalytic materials and processes designed specifically for the efficient neutralization of hazardous nitrogen mustard agents. This structural interpretation should not be regarded as a quantitative toxicological conclusion, because direct toxicity testing or validated QSAR-based toxicity estimation of the intermediates was not performed in the present study.

To clearly illustrate the comparative degradation efficiencies and reinforce the mechanistic implications, a comprehensive summary of photocatalytic degradation results for all CWAs studied in this work (GD, HD, HN1, and HN2) is provided in Figure 7.

### 3.3. Advantages and Limitations of TiO_2_ Photocatalytic Systems

TiO_2_-based photocatalytic systems present numerous advantages as an environmentally sustainable and efficient solution for decontaminating CWAs. Firstly, the use of solar energy, an abundant and renewable resource, makes photocatalytic processes highly attractive from both environmental and economic perspectives [5,11,21]. Unlike traditional chemical neutralization methods, in ideal cases, photocatalytic oxidation can proceed toward mineralized products such as carbon dioxide, water, and inorganic salts; however, complete mineralization was not demonstrated in the present study [13,30,31].

Moreover, TiO_2_ photocatalysts offer high chemical stability, non-toxicity, and cost-effectiveness, which are critical factors when considering large-scale or long-term field deployment [1,2,34]. The passive and continuous nature of photocatalytic surfaces, which require minimal operational intervention beyond initial catalyst installation, further enhances their practicality for military, industrial, and civilian applications [15,18]. Our findings reinforce these advantages, particularly highlighting the potential for passive, continuous self-decontamination in various application scenarios.

Despite these considerable advantages, several limitations inherent to TiO_2_ photocatalytic systems must be acknowledged. One primary limitation is the dependence on adequate photon energy (UV or visible light) to initiate and sustain the catalytic process effectively [6,8,17]. Under conditions of low or intermittent solar irradiation, degradation rates significantly decrease, as clearly shown in Figure 7, potentially compromising decontamination effectiveness in real-world scenarios.

Another notable limitation is the relatively slower reaction kinetics compared to conventional chemical or thermal degradation methods. Photocatalytic degradation often requires extended exposure times to achieve substantial degradation efficiencies, potentially limiting its suitability for emergency scenarios requiring immediate neutralization of highly toxic agents [3,33,36]. Furthermore, catalyst deactivation through fouling, accumulation of degradation byproducts, or physical abrasion may reduce photocatalytic efficiency over time, necessitating periodic maintenance or replacement [32].

Recent research efforts have focused on addressing these limitations by enhancing the photocatalytic performance of TiO_2_ through doping, composite formation, and surface modification techniques. For instance, doping TiO_2_ with metals such as silver, copper, or iron, or forming composite structures with graphene or other semiconductor materials, has shown promising results in improving visible-light absorption and overall catalytic efficiency [11,12,29,31,36]. These approaches, however, introduce additional complexities related to cost, scalability, and long-term stability, which require thorough evaluation before practical implementation.

In summary, while TiO_2_ photocatalytic systems offer clear advantages regarding sustainability, environmental friendliness, and cost-effectiveness, their practical utility may be constrained by operational limitations such as dependence on solar illumination and relatively slower kinetics. Future research should therefore aim at overcoming these limitations, focusing on optimizing photocatalytic materials and processes to enhance degradation efficiency, broaden applicability, and ensure robust performance under diverse field conditions.

### 3.4. Implications for Practical Field Implementation and Future Directions

While the photocatalytic degradation performance demonstrated in this study suggests strong potential for real-world applications, practical field implementation of TiO_2_-based decontamination systems requires careful consideration of several operational and environmental factors.

One major challenge in field deployment is the variability of solar irradiation, which directly influences photocatalytic efficiency. As highlighted by the substantial performance drop under dark conditions, as shown in Figure 7, field systems must be designed to either maximize light capture or incorporate supplemental artificial light sources for operation under low-light or nighttime conditions [5,11,29]. Developing photocatalysts with extended activity under visible light or integrating hybrid systems that combine photocatalysis with alternative degradation mechanisms could enhance reliability in diverse operational environments [12,31].

Another critical consideration is the mechanical durability and environmental stability of photocatalytic coatings or composites intended for field use. Surfaces may be exposed to harsh environmental conditions, including temperature fluctuations, abrasion, and chemical fouling, all of which could compromise long-term catalytic performance [32]. Strategies such as embedding TiO_2_ nanoparticles within robust polymer matrices or developing self-healing photocatalytic surfaces are promising approaches that warrant further investigation [25,33].

From an operational perspective, scaling up photocatalytic decontamination from laboratory conditions to large-area field applications introduces challenges in catalyst deployment, uniformity of coating, maintenance requirements, and cost-effectiveness. Addressing these issues will require interdisciplinary collaboration among materials scientists, chemical engineers, and field operation experts to optimize system designs tailored to specific deployment scenarios.

Future research should also focus on the development of multi-functional photocatalytic materials capable of degrading a broad spectrum of chemical and biological threats, thereby enhancing the overall resilience and versatility of field-deployable decontamination systems. In addition, life cycle assessment (LCA) and environmental impact analyses of photocatalytic technologies are needed to ensure that large-scale implementation does not introduce unintended ecological consequences [17,18,36].

In summary, while TiO_2_-based photocatalysis holds considerable promise for CWA decontamination, realizing its full potential in practical field applications will require technological advancements that address light-dependence, material durability, system scalability, and holistic environmental safety.

Continued multidisciplinary research and pilot-scale field testing are essential next steps toward bridging the gap between laboratory success and operational reality.

## 4. Materials and Methods

### 4.1. Chemicals and Photocatalyst Preparation

TiO_2_ (purity 99.5%, Sigma-Aldrich, St. Louis, MO, USA), consisting of a mixed phase of rutile and anatase (particle size < 100 nm), was used as the photocatalyst [11,12]. The reaction solvent was prepared by mixing acetonitrile (99%) with 1% water to achieve polarity suitable for photocatalytic degradation reactions [14]. Chemical warfare agents (GD, HD, HN1, and HN2) were synthesized by an official institute authorized by the Organization for the Prohibition of Chemical Weapons (OPCW) for the synthesis, research, and storage of CWAs, and were handled under strictly regulated laboratory conditions in compliance with OPCW guidelines. The commercial TiO_2_ powder corresponds to a mixed-phase rutile/anatase formulation with a nominal primary particle size below 100 nm, as specified by the supplier; the as-received powder was used without further treatment. Independent morphological characterization (SEM/TEM) and specific surface area determination (BET) were not carried out as part of the present proof-of-concept evaluation under live-agent constraints; reference characterization values for this commercial reagent are consistent with anatase/rutile mixed-phase TiO_2_ photocatalysts widely employed in the literature. A dedicated catalyst-characterization block is foreseen for the follow-up study.

### 4.2. Photocatalytic Reaction Setup

Reaction mixtures were prepared by dissolving each CWA to a final concentration of 200 ppm in the acetonitrile–water solvent. TiO_2_ was added at 1% (*w*/*v*) to the reaction mixtures. The samples were divided into three groups: (1) sunlight exposure with TiO_2_ (Sun + TiO_2_), (2) sunlight exposure without TiO_2_ (Sun only), and (3) dark control with TiO_2_ (Dark).

Each reaction mixture (5 mL) was transferred into a transparent glass vial (5 mL capacity). Sunlight-exposed samples were placed outdoors under direct sunlight, whereas dark control samples were wrapped in aluminum foil and stored indoors in a dark box.

The photocatalytic reactions were conducted under natural sunlight conditions, with solar irradiance ranging approximately from 300 to 800 W·m^−2^ depending on the time of day. Although the irradiance was not continuously recorded, all experiments were performed between 10:00 AM and 4:00 PM to ensure consistent and sufficient solar exposure. The reported irradiance interval (300–800 W·m^−2^) represents the variability of natural sunlight during the experimental window (10:00–16:00) and was estimated rather than continuously logged; the integrated daily irradiation dose and the ultraviolet fraction were not recorded and are acknowledged as a limitation of the present proof-of-concept study. Experiments for all four agents and their corresponding controls were performed in parallel on the same day in adjacent vials to ensure identical solar exposure. Ambient temperature ranged from 23 to 31 °C, and only clear-to-lightly cloudy days were used; experimental dates and weather records are retained at the OPCW-designated facility and available upon regulated request.

Photocatalytic reactions were carried out for 180 and 360 min for GD and HD. For HN1 and HN2, additional time points at 10, 30, and 60 min were included to monitor detailed degradation kinetics.

At each designated time point, 50 mg of anhydrous magnesium sulfate (MgSO_4_) was added to each vial to remove residual water. After gentle shaking and standing for 1 min, the suspension was filtered through a syringe equipped with a membrane filter to eliminate TiO_2_ particles and MgSO_4_. Reaction conditions and sample compositions are summarized in Table 3.

Photocatalytic degradation progress was monitored at specific intervals (10, 30, 60, 180, and 360 min).

The present experiment was conducted as a screening-scale proof-of-concept study under OPCW-regulated live-agent constraints. The reported values are therefore presented as condition-specific degradation estimates obtained under the described experimental window rather than as mean ± standard deviation values from a fully powered replicate study. A future controlled-irradiation campaign will be required to obtain replicate statistics, recovery values, internal-standard-based quantitation, and limit-of-detection and limit-of-quantitation parameters. No extrapolated standard deviations or validation parameters were introduced into the present manuscript.

### 4.3. Analytical Methods

Samples were dehydrated using magnesium sulfate (MgSO_4_, 50 mg), vortexed briefly, and filtered through a syringe filter. Chromatographic analysis of degradation products utilized gas chromatography with flame photometric detection (GC-FPD) for GD and HD, and gas chromatography with flame ionization detection (GC-FID) for HN1 and HN2, as summarized in Table 4 [22,23].

### 4.4. Mechanistic Analysis by HR-MS

HR-MS analysis was performed using a Thermo Orbitrap-based mass spectrometer (Q Exactive Plus; Thermo Fisher Scientific, Waltham, MA, USA) coupled with a Vanquish liquid chromatography system (Thermo Fisher Scientific, Waltham, MA, USA). This setup enabled high-resolution detection of intermediate species formed during the photocatalytic degradation of HN-series agents. The analysis was carried out to obtain qualitative mechanistic information on the transformation of HN-series agents, with data acquired under standard operating conditions. Mass spectra were acquired in positive-mode electrospray ionization (ESI+) over an *m*/*z* range of 50–750 at a resolving power of 70,000 (FWHM at *m*/*z* 200). Because the present study was not designed as a full HR-MS/MS intermediate-identification campaign under live-agent constraints, complete extracted-ion chromatograms, product-ion spectra, and a validated intermediate-assignment table are not provided. These analyses are identified as priorities for future mechanistic confirmation.

### 4.5. Kinetic and Statistical Analysis

The photocatalytic degradation behavior of chemical warfare agents was evaluated using an apparent pseudo-first-order model. The kinetic model follows the general first-order rate equation:

*ln(C_t_/C*_0_*) = −k_app_ · t*

where *C_t_* is the concentration of the agent at time *t*, *C*_0_ is the initial concentration, and *k_app_* is the apparent rate estimate. For comparative evaluation, endpoint-derived apparent rate estimates were calculated from the 360 min degradation efficiencies using *k_app_ = −ln(C_t_/C*_0_*)/t*. No dedicated statistical software was used; the endpoint-derived apparent rate estimates were calculated directly from the equation above.

### 4.6. Safety and Compliance

All experiments involving live chemical warfare agents were conducted in an OPCW-designated laboratory by trained and authorized personnel in accordance with approved standard operating procedures and institutional risk-management protocols. All experimental manipulations were performed using appropriate engineering controls, including ventilated containment systems and restricted-access work areas, to minimize personnel exposure. Task-appropriate personal protective equipment, including protective laboratory clothing, chemically resistant gloves, and eye and face protection, was worn throughout the study, with respiratory protection used when required by the site-specific risk assessment. Contaminated consumables, residual agents, filtrates, and decontamination residues were segregated, collected in properly labeled compatible containers, and managed through approved hazardous-waste disposal procedures in accordance with institutional requirements, national regulations, and OPCW guidelines. Emergency response and personnel decontamination procedures were established and maintained for all operations involving live agents.

## 5. Conclusions

This study provides a laboratory-scale proof-of-concept demonstration of TiO_2_-mediated solar photocatalytic parent-agent degradation for actual CWAs, including GD, HD, HN1, and HN2. Substantial decreases in parent-agent signals were observed, with the highest apparent degradation observed for HN2 (93% after 6 h) under the tested natural-sunlight conditions. The comparison between HN1 and HN2 suggests that structural differences may influence photocatalytic susceptibility, although the proposed steric and ROS-mediated explanations for the higher apparent reactivity of HN2 remain working hypotheses rather than experimentally confirmed mechanisms. The proposed steric and ROS-mediated explanations for the comparative reactivity of HN2 versus HN1 remain working hypotheses rather than experimentally confirmed mechanisms.

HR-MS analysis provided qualitative mechanistic information consistent with degradation pathways involving sequential dechlorination, hydrolysis, and oxidative cleavage; a complete intermediate-assignment confirmation is identified as future work. The use of actual CWAs rather than simulants significantly strengthens the practical relevance of this study and fills an important gap in the current literature.

The results underscore the potential of TiO_2_-based photocatalytic systems as sustainable, passive decontamination strategies for chemical threat mitigation. Nevertheless, the practical implementation of such systems requires addressing key challenges, including dependence on solar irradiation, relatively slow reaction kinetics, and the durability of photocatalytic coatings under field conditions.

The present study is subject to important limitations. The experiments were performed in homogeneous acetonitrile/water (99:1) suspension under variable natural sunlight, and the current dataset does not include full replicate statistics, continuous UV-dose logging, complete analytical validation, quantitative toxicity assessment of intermediates, ROS-scavenger experiments, or full HR-MS/MS intermediate confirmation. Therefore, the results should not be interpreted as direct evidence of complete detoxification, mineralization, or field performance on coatings, textiles, or operational surfaces.

Future work should therefore focus on three priorities aligned with the limitations identified above: (i) controlled-irradiation kinetics with continuous logging of integrated dose and UV fraction, supported by full replicate statistics and validated quantitative analysis (LOD/LOQ, recovery, internal standards); (ii) ROS-scavenger and spin-trap experiments together with a complete HR-MS/MS intermediate-assignment campaign, and quantitative toxicological evaluation of the intermediates (in silico QSAR and in vitro cytotoxicity); (iii) catalyst characterization (SEM/TEM, BET, phase composition) and coated-substrate or field-relevant surface studies. These follow-up studies will be necessary to convert the present proof-of-concept findings into a robust and practically deployable photocatalytic decontamination platform.

Overall, this study contributes foundational knowledge towards the development of environmentally friendly, solar-driven decontamination technologies capable of addressing a broad spectrum of chemical threats in military, industrial, and civilian settings. This study should be understood as a laboratory-scale proof-of-concept demonstration; broader operational claims are reserved for the controlled follow-up campaigns outlined above.

## Figures and Tables

**Table 1:** Photocatalytic degradation results of chemical warfare agents (CWAs; GD and HD) under different conditions after 6 h.

CWA	Condition	Residual Concentration (%)	Degradation Efficiency (%)
GD	Dark	97.1	2.9
GD	Sunlight only	93.0	7.0
GD	TiO_2_ + Sunlight	40.0	60.0
HD	Dark	100.0	0.0
HD	Sunlight only	90.2	9.8
HD	TiO_2_ + Sunlight	37.3	62.7

**Table 2:** Endpoint-estimated apparent rate constants for the TiO_2_-mediated photocatalytic degradation of CWAs under sunlight.

Agent	Reaction Time (min)	Degradation Efficiency (%)	C/C_0_	Apparent Rate Constant, k (min^−1^)	k (h^−1^)
GD	360	60.0	0.400	2.55 × 10^−3^	0.153
HD	360	62.7	0.373	2.74 × 10^−3^	0.164
HN1	360	76.0	0.240	3.96 × 10^−3^	0.238
HN2	360	93.0	0.070	7.38 × 10^−3^	0.443

**Table 3:** Experimental conditions for photocatalytic reactions.

Sample ID	CWA (200 ppm)	TiO_2_	Solar Exposure
a	HN1	Yes	Yes
b	HN1	No	Yes
c	HN1	Yes	No
d	HN2	Yes	Yes
e	HN2	No	Yes
f	HN2	Yes	No
g	GD	Yes	Yes
h	GD	No	Yes
i	GD	Yes	No
j	HD	Yes	Yes
k	HD	No	Yes
l	HD	Yes	No

**Table 4:** Gas chromatography analytical conditions.

Parameter	Condition
GC instrument	Agilent 7890 (Agilent Technologies, Santa Clara, CA, USA)
Column	DB-5 (30 m × 0.32 mm × 0.25 µm; Agilent Technologies, Santa Clara, CA, USA)
Oven temperature	40 °C (1 min), 10 °C/min to 250 °C (1 min hold)
Detector temperature	250 °C
Carrier gas	N_2_
Air flow	95 mL/min

## Data Availability

The data supporting the findings of this study are available from the corresponding author upon reasonable request, subject to institutional safety, security, and regulatory restrictions related to live CWA experiments.

## Abbreviations

The following abbreviations are used in this manuscript:

CWA	Chemical Warfare Agent
OPCW	Organization for the Prohibition of Chemical Weapons
HR-MS	High-Resolution Mass Spectrometry
GC-FPD	Gas Chromatography with Flame Photometric Detection
GC-FID	Gas Chromatography with Flame Ionization Detection
